# The Moderating Role of Confucian Coping in the Job Demands–Resources Model in Chinese Tertiary Hospitals

**DOI:** 10.1155/jonm/1459619

**Published:** 2026-03-31

**Authors:** Mengzhu Deng, Aristides I. Ferreira

**Affiliations:** ^1^ Department of Human Resource Management, Guizhou Provincial People’s Hospital, No. 83 Zhongshan East Road Nangming District, Guiyang, Guizhou, China, 5055.cn; ^2^ Iscte-Instituto Universitário de Lisboa (Iscte-IUL) & Business Research Unit (BRU-IUL), Avenida das Forças Armadas, Lisboa, 1649-026, Portugal

**Keywords:** Confucian coping, healthcare professionals, job burnout, job demands–resources theory, job engagement, moderating effect

## Abstract

**Background:**

Medical staff in Chinese tertiary hospitals experience excessive workloads, increasing burnout vulnerability. Traditional cultural resources may influence their job attitudes, but this area remains unexplored.

**Purpose:**

Based on the job demands–resources model, this study investigates how Confucian coping, as a personal culture resource, moderates the relationships among job demands, resources, engagement and burnout in Chinese medical staff.

**Methods:**

Using an online self‐administered survey, we collected data from 1653 medical staff members across 14 tertiary hospitals in China. Structural equation modelling was used to test the hypothesised moderating pathways.

**Results:**

Confucian coping demonstrated a significant positive moderating effect on the job resources–job engagement relationship and a significant negative moderating effect on the job demands–job burnout relationship.

**Conclusion:**

Confucian coping serves as a significant personal resource for medical staff, mitigating burnout by buffering job demands and enhancing engagement by amplifying job resources.

**Originality:**

By employing empirical analysis with the job demands–resources model, this study unravels how medical staff draw on Confucian coping functions and provides a new theoretical perspective for further study of the influence of cultural and psychological factors.

## 1. Introduction

In China, tertiary hospitals provide specialised services, including the diagnosis of complex diseases, advanced surgical interventions and intensive care, whereas primary and secondary hospitals are smaller and offer only basic medical support [1]. Although tertiary hospitals constitute only 8.4% of the total hospital capacity, they provide over half of the outpatient and inpatient services. This is due to underdeveloped hierarchical referral systems. The bed utilisation rate in tertiary hospitals is reported as 97.5%, compared with the national average of 83.6%. Healthcare professionals in tertiary hospitals have excessive workloads, becoming prone to burnout. This is particularly true for nurses, who form the clinical backbone of healthcare teams. Beyond these, they face unique challenges such as managing high expectations and distrust from patients’ families [2], power hierarchies between doctors and nurses [3], career development bottlenecks [4], and the emotional labour of upholding societal harmony [5]. Similarly, doctors bear the ultimate diagnostic and treatment responsibility, whereas pharmacists face different pressures related to supply chain management [6].

Previous studies on Chinese medical staff job engagement and burnout have explored several influencing factors, such as the impact of personality [7], work intensity [8], leadership style [9] and organisational culture [10]. However, national cultural factors remain underexplored. Research on coping with stress, which ignores cultural differences, may lead to misguided conclusions [11]. Hence, this study focuses on Chinese Confucian cultural factors.

Unlike Western cultures and religions, Confucian culture emphasises worldly rationalism, encourages people to follow the laws of nature, and cultivates personal character and behaviour [12]. It profoundly influences Chinese people’s characteristics and coping styles [13] and provides a framework for nurses to manage their personal emotions and maintain professional decorum under extreme stress [14]. The core virtue of ‘*Ren*’ (仁, benevolence) resonates deeply with the nursing ethos of care and compassion [15], and ‘*Li*’ (礼, propriety) aligns with the need for procedural adherence and respectful communication. Confucian culture also influences medical education in China. For instance, mottos from medical colleges frequently invoke Confucian terms such as ‘*De*’ (德, virtue), ‘*Xue*’ (学, learning) and ‘*Jing*’ (精, excellence) [16]. These values are also included in the Chinese version of the ‘Oath of Medical Students’ released by the State Education Commission of China and the ‘Declaration of Chinese Doctors’ released by the Chinese Medical Doctor Association (CMDA) [17]. Hence, understanding how medical staff draw on Confucian coping strategies in their daily stressful medical work is important.

To explore these complex relationships, we employ the job demands–resources (JD–R) model as our theoretical framework [18]. The JD–R model defines two types of job characteristics: job demands and resources [19]. Job demands refer to the physical, psychological, social, or organisational aspects of a job that require sustained physical or psychological effort or skills and therefore have certain physiological or psychological costs [20]. Job resources are aspects that support the achievement of work objectives; mitigate job demands and their accompanying costs; and foster personal growth, learning and development [19]. A distinctive feature of the JD–R model is its flexibility, allowing demands and resources to adapt to specific occupational contexts [21].

The following sections explore the theoretical rationale and contributions, extending the JD–R model to the health sector [22] and integrating personal resources in the form of Confucian coping strategies. This study highlights the importance of certain Eastern values that may also influence other contexts and geographical regions [23]. It also makes a relevant contribution to the JD–R model by understanding its plasticity in domains linked to cultural personal resources as moderators that strengthen the role of the demands and resources that healthcare workers encounter in their work.

## 2. Theoretical Background and Hypotheses

### 2.1. The Influence of Job Demands on Job Burnout and Engagement

In the healthcare context, numerous factors contribute to significant job demands. Work overload is a prevalent and primary predictor of burnout among medical staff [24]. Chinese healthcare professionals frequently work extended hours under a poorly implemented annual leave system. They also undertake frequent night or weekend call duties, exacerbating their exhaustion [25]. Such sustained demands deplete both mental and physical resources, contributing significantly to health impairment and burnout [26]. Additionally, work overload intensifies work–home conflict. This is particularly evident among nurses who are predominantly female and often bear most of the domestic and childcare responsibilities within traditional Chinese family structures. Tensions between professional duties and family obligations are then exacerbated. Work–home conflict has been shown to be a critical determinant of job satisfaction and well‐being. It correlates positively with turnover intention and negatively with engagement [27]. Research stress is a noteworthy demand that is uniquely amplified by the academic medicine context in China. In tertiary hospitals, professional title evaluation, which is closely tied to salary, pension and career advancement, heavily emphasises publication output. This pressure necessitates a demanding dual role of clinical work by day and academic writing by night, creating a salient stressor, particularly among junior doctors [28]. Furthermore, continuous vigilance, rapid decision‐making and multitasking amid frequent interruptions create a high cognitive load. This mental demand constitutes another crucial dimension of job demands, particularly for nurses managing complex patient needs in dynamic clinical settings [29]. Finally, Chinese medical staff frequently face high expectations and occasionally distrust from patients’ families [30]. These can manifest as verbal confrontations or even workplace violence, which are associated with increased depressive symptoms [31] and elevated risks of burnout, emotional exhaustion and anxiety disorders among medical staff [32]. Together, work overload, work–home conflict, research stress, mental demand and workplace violence characterise China’s healthcare sector as a challenging occupational landscape. Hence, this study incorporates them as constitutive elements of job demands. Thus, we propose the following hypotheses:

 H1: Job demands (work overload, work–home conflict, research stress, mental demand and workplace violence) are positively correlated with job burnout.
 H2: Job demands (work overload, work–home conflict, research stress, mental demand and workplace violence) are negatively correlated with job engagement.

### 2.2. The Influence of Job Resources on Job Burnout and Engagement

In the context of China’s healthcare system, which is characterised by a hierarchical structure, high workload, and a collectivist cultural background, job resources play a critical role in mitigating occupational stress and enhancing employee well‐being. The perception of achievable career pathways within institutional constraints is crucial for maintaining motivation [33]. Hence, perceived career opportunities are salient, particularly for nurses who often face limited upward mobility. Person–job fit is another key resource reflecting the alignment between healthcare workers’ personal values and organisational roles. A strong fit is associated with greater job satisfaction and reduced turnover intention, especially among healthcare professionals who must balance emotional labour with technical responsibilities [34]. Organisational status perception also significantly influences medical professionals’ sense of value and belonging. In China’s prestige‐sensitive medical environment, feeling respected within one’s organisation enhances commitment and mitigates burnout [35]. Interpersonal relationships are another critical resource in the Chinese healthcare system’s team‐orientated culture. Harmonious relationships with colleagues, supervisors and patients in high‐stakes and interdependent work settings can significantly buffer workplace stress [36]. Finally, organisational justice—perceptions of fairness in decision‐making, reward distribution, and respect from authorities—is a fundamental protective factor [37]. In a system characterised by structural power imbalances and high‐performance pressure, organisational justice is associated with higher engagement and lower burnout among medical occupational groups [38, 39]. This study incorporates perceived career opportunities, person–job fit, organisational status perception, interpersonal relationships, and organisational justice as job resources. Considering the above, we propose the following hypotheses:

 H3: Job resources (perceived career opportunities, person–job fit, organisational status perception, interpersonal relationships and organisational justice) are negatively correlated with job burnout.
 H4: Job resources (perceived career opportunities, person–job fit, organisational status perception, interpersonal relationships and organisational justice) are positively correlated with job engagement.

### 2.3. The Moderating Effect of Confucian Coping as a Personal Resource

The JD–R model has been applied to various medical contexts [40, 41]. While personal resources, such as personality traits and self‐efficacy, have been incorporated, the model has largely overlooked cultural coping mechanisms. This presents a theoretical gap, particularly in non‐Western contexts, where cultural frameworks fundamentally shape stress appraisal and responses [42]. Confucian coping has received increasing attention in recent years as China has developed; it is defined as the application of Confucian principles to navigate adversity and setbacks [43] by considering adversity as an opportunity for growth through introspection, self‐cultivation and active engagement [44]. However, previous studies have often used it as an independent variable rather than a moderating variable [45]. In this study, we adopt Confucian coping as a key moderating variable. We use Jing’s scale, which comprises four distinct dimensions: ‘Optimism in Adversity’, ‘Viewpoints on Fate’, ‘Responsibility as Human Beings’ and ‘The Role of Adversity in Individual Growth’. These dimensions may help medical staff, particularly nurses, reinterpret and manage their job demands.

For instance, ‘Optimism in Adversity’ and ‘The Role of Adversity in Individual Growth’ enable nurses to reframe challenging situations, such as dealing with aggressive patients or complex clinical cases, as opportunities for professional growth rather than threats, thereby reducing emotional exhaustion and preserving engagement [46]. ‘Viewpoints on Fate’ may help medical staff accept medically uncontrollable outcomes without guilt or frustration [47]. ‘Responsibility as Human Beings’ reinforces the sense of moral duty that motivates nurses to provide compassionate care even under high workloads, thereby enhancing the perceived meaningfulness of their work [48]. Considering the above, we propose the following hypotheses:

 H5: Confucian coping moderates the influence of job demands on job burnout. The influence of job demands on job burnout weakens when the degree of personal Confucian coping is high.
 H6: Confucian coping moderates the influence of job resources on job engagement. The influence of job resources on job engagement strengthens when the degree of personal Confucian coping is high.

**FIGURE 1:**
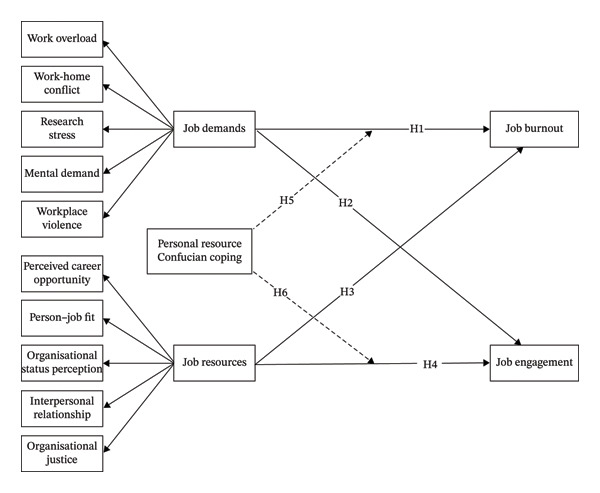
Conceptual model.

Based on the above research hypotheses, this study’s conceptual model is shown in Figure 1.

## 3. Methods

### 3.1. Study Design and Participants

This study conducted a cross‐sectional survey using a stratified three‐stage sampling method to select medical staff working in tertiary public hospitals in Guizhou Province from February to April 2022. Eligible participants were full‐time medical staff (doctors, nurses, pharmacists and technicians) with at least 1 year of work experience in the selected hospitals. Temporary and administrative personnel were excluded from the study. Questionnaires completed in less than 1 minute or that had missing or insufficient responses were considered invalid and were not accepted.

### 3.2. Samples

**FIGURE 2:**
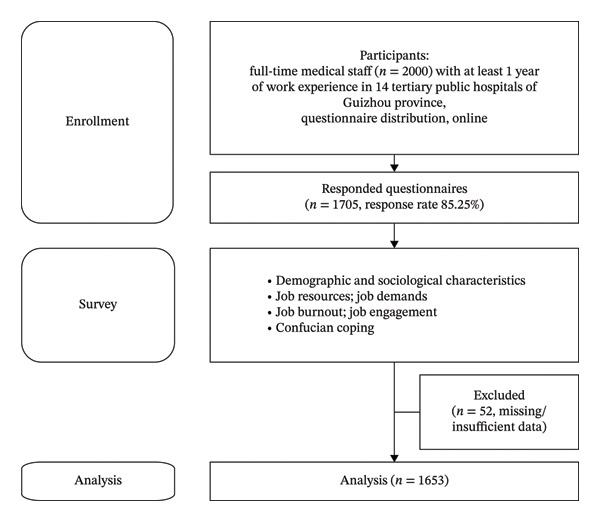
Study sampling.

Based on the *China Health Statistics Yearbook 2021* [49], which reported 100,446 urban healthcare technicians in Guizhou Province, the minimum sample size was determined using a finite population sampling formula. With a confidence level of 95%, margin of error of 5%, and proportion estimate set at 0.5, the calculated minimum sample size was 385. To account for potential sampling errors and nonresponse, 2000 questionnaires were distributed, yielding 1705 completed questionnaires (response rate: 85.25%). After excluding 52 responses owing to missing or insufficient responses, 1653 were analysed and considered an adequate sample size (Figure 2).

### 3.3. Data Collection

This study employed a stratified three‐stage sampling approach to enhance representativeness and capture the structural diversity within the healthcare workforce in Guizhou Province, China. First, 14 tertiary public hospitals were systematically selected from 34 eligible hospitals across all nine prefectures. Proportional representation (≈40% per prefecture) was used to account for regional variability in resource distribution, patient demographics and institutional size. This step aimed to mitigate geographic bias and improve sample generalisability to diverse tertiary care settings in the province. Second, each hospital was proportionally represented in the sample based on the total number of technical health staff members, yielding a planned sample of 2000 participants. This approach ensured that larger hospitals, which typically serve more patients and employ more staff, contributed more respondents to the sample, thereby improving proportionality and reducing oversampling from smaller institutions. Third, within each hospital, participants were stratified into three professional categories using provincial workforce ratios (doctors:nurses:pharmacists/technicians = 1:1.84:0.33), derived from the *China Health Statistics Yearbook 2021* [49]. In total, the sample comprised 640 doctors, 1160 nurses, and 200 pharmacists/technicians, reflecting the actual composition of Guizhou’s tertiary hospital workforce. Random selection from employee registries was conducted using computer‐generated lists, with further stratification by clinical department, where feasible. While this stratified approach improves sample representativeness across key structural and professional dimensions, potential biases may arise from the exclusion of nonpublic hospitals, possible underrepresentation of very small departments, and differences in response rates across professional groups.

The survey was administered electronically through Wenjuanxing, a widely used online questionnaire platform in China. Personnel managers at each participating hospital distributed the survey link (via QR code) to the eligible staff. To prevent duplicate submissions, the responses were restricted to one per IP address.

### 3.4. Ethical Considerations

This study was approved by the Ethics Committee of the Guizhou Provincial People’s Hospital (Approval Number: 2021‐89). Before the investigation, all participants were informed of the study purpose, voluntary nature of participation, confidentiality of data, and right to withdraw at any time. All data were anonymised according to the ICMJE guidelines.

### 3.5. Measures

The questionnaire comprised six parts: personal information, job demands, job resources, job burnout, job engagement and personal resources. All instruments were administered in Chinese, with previously validated versions demonstrating satisfactory reliability.

#### 3.5.1. Demographic Data

This study employed a self‐designed general information questionnaire to investigate sociodemographic characteristics such as gender, education level, years of work experience, job position, professional title, weekly working hours, night‐shift frequency per week and employment method.

#### 3.5.2. Job Burnout

Job burnout was measured using the seven‐item Work‐Related Burnout scale from the Copenhagen Burnout Inventory [50]. Participants were asked to indicate how frequently they experienced each feeling in relation to their current hospital job. Six response options were available, ranging from 1 (*never*) to 6 (*always*). The total scores on this scale range from 7 to 42, with higher scores indicating higher burnout [51]. This study’s Cronbach’s alpha coefficient was 0.894.

#### 3.5.3. Job Engagement

Job engagement was assessed using the nine‐item Utrecht Work Engagement Scale (UWES‐9) developed by Schaufeli [52], which is divided into three subscales: Vigour (three items), Dedication (three items) and Absorption (three items). The statements were presented on a six‐point Likert scale ranging from 1 (*never*) to 6 (*always*). The total scores range from 9 to 54, with a higher score indicating better job engagement. This study’s Cronbach’s alpha coefficients for vigour, dedication, absorption and overall job engagement were 0.853, 0.863, 0.855 and 0.895, respectively.

#### 3.5.4. Job Demands

Job demands were evaluated using 19 items across five dimensions: workload (three items) [53], work‐home conflict (three items) [54], research stress (three items) [55], mental demand (three items) [53], and workplace violence (seven items) [56]. Participants were asked to evaluate how well the question corresponded to their actual situation and select the corresponding item score, with six response options ranging from 1 (*totally disagree*) to 6 (*totally agree*). The total scores on the job demands scale ranged from 19 to 114, with a higher score suggesting higher job demands. In this study’s formal survey, the scale had good internal consistency, with Cronbach’s alpha coefficients for workload, work–home conflict, research stress, mental demand, workplace violence, and overall job demands being 0.828, 0.860, 0.870, 0.792, 0.943 and 0.917, respectively.

#### 3.5.5. Job Resources

Job resources were assessed using a questionnaire that included 18 items grouped into five factors: perceived career opportunities (three items) [57], person–job fit (four items) [58], organisational status perception (four items) [59], interpersonal relationships (four items) [60], and organisational justice (three items) [61]. Participants were asked to evaluate how well each question corresponded to their actual situation and choose the corresponding item score, with six response options ranging from 1 (*totally disagree*) to 6 (*totally agree*). Total scores on the job resources scale ranged from 18 to 108, with higher values reflecting higher job resources. This study’s Cronbach’s alpha coefficients for perceived career opportunities, person–job fit, organisational status perception, interpersonal relationships, and organisational justice were 0.833, 0.867, 0.781, 0.84 and 0.831, respectively. The Cronbach’s alpha coefficient for the overall job resources questionnaire was 0.915.

#### 3.5.6. Confucian Coping

Personal resources (Confucian coping) were assessed using a 15‐item Chinese version of the Confucian Coping Questionnaire [43]. It has four dimensions, including: ‘Optimism in Adversity’ (three items), ‘Viewpoints on Fate’ (three items), ‘Responsibility as Human Beings’ (four items) and ‘The Role of Adversity in Individual Growth’ (five items). These items were scored on a six‐point scale ranging from 1 (*totally disagree*) to 6 (*totally agree*). Total scores ranged from 15 to 90, with higher values reflecting higher levels of Confucian coping [62]. This study’s Cronbach’s alpha coefficient was 0.899.

### 3.6. Strategy of Analysis

We employed structural equation modelling (SEM) in Mplus 8.3 to examine the hypothesised relationships. SEM offers distinct advantages over traditional regression methods by enabling the simultaneous estimation of multiple pathways among latent variables. This accounts for measurement errors and provides comprehensive model‐fit indices for the theoretical evaluation. Before the analysis, Harman’s test was conducted on all scale items; the results showed that the percentage of variance explained by the first factor that did not rotate in the principal component factor analysis was 28.048%. As this percentage was less than 50%, no serious common method deviation existed in the questionnaire [63]. Initially, the SEM analysis evaluated a measurement model to confirm the psychometric properties of the constructs. This was followed by testing a structural model to examine both the direct effects of job demands and resources on burnout and engagement and the moderating role of Confucian coping within a latent variable interaction framework. Statistical significance was set at *p* < 0.05.

## 4. Results

### 4.1. Demographic Characteristics

The final sample comprised 1653 medical staff. Overall, the sample was predominantly female (76.29%) and fell largely within the 30–40 age group (54.93%). In terms of occupational distribution, nurses constituted the majority (58.08%), followed by doctors (31.28%). Regarding workplace seniority, 28.86% of the participants had worked for 10–15 years and 25.71% had worked for 5–9 years. The weekly working hours of more than half the participants (53.84%) ranged from 40 to 49 h. Further details on the samples are provided in Table 1.

**TABLE 1:** Demographic information about the participants (*n* = 1653).

Items	Categories	*N*	Percent (%)
Gender	Female	1261	76.29
	Male	392	23.71

Age	Under 30	419	25.35
	30–40	908	54.93
	41–50	236	14.28
	51–60	88	5.32
	Above 60	2	0.12

Education level	High school degree	10	0.60
	Bachelor’s degree or tertiary college	1384	83.73
	Master’s degree	213	12.89
	Doctoral degree	46	2.78

Years of work experience	Less than 5 years	396	23.96
	5–9 years	425	25.71
	10–15 years	477	28.86
	16–20 years	153	9.26
	More than 20 years	202	12.22

Position	Doctor	517	31.28
	Nurse	960	58.08
	Technician	133	8.05
	Pharmacist	43	2.60

Professional title	No title	112	6.78
	Primary professional title	679	41.08
	Intermediate professional title	575	34.79
	Subsenior professional title	206	12.46
	Senior professional title	81	4.90

Weekly working hours	Less than 40 h	208	12.58
	40–49 h	890	53.84
	50–59 h	286	17.30
	60–69 h	137	8.29
	70–79 h	70	4.23
	More than 80 h	62	3.75

Night‐shift frequency per week	None	653	39.50
	1‐2 times	788	47.67
	3‐4 times	182	11.01
	More than 5 times	30	1.81

Employment method	Regular	848	51.30
	Contract	805	48.70

Total		1653	100.0

### 4.2. Descriptive Statistics

Descriptive statistics and correlations for the study scales are shown in Table 2. All correlations were in the hypothesised direction.

**TABLE 2:** Descriptive statistics and correlations.

Characteristics	M	SD	1	2	3	4	5	6	7	8	9	10	11	12	13
Gender	1.763	0.425	1												
Education level	2.178	0.463	−0.249^∗∗^	1											
Years of work experience	2.601	1.280	0.045	−0.071^∗∗^	1										
Position	1.581	0.494	0.515^∗∗^	−0.414^∗∗^	−0.017	1									
Professional title	2.676	0.948	−0.184^∗∗^	0.327^∗∗^	0.634^∗∗^	−0.374^∗∗^	1								
Weekly working hours	2.490	1.171	−0.284^∗∗^	0.288^∗∗^	−0.093^∗∗^	−0.376^∗∗^	0.118^∗∗^	1							
Night‐shift frequency per week	1.751	0.718	−0.078^∗∗^	0.030	−0.271^∗∗^	−0.064^∗∗^	−0.196^∗∗^	0.207^∗∗^	1						
Employment method	1.513	0.500	0.234^∗∗^	−0.349^∗∗^	−0.400^∗∗^	0.450^∗∗^	−0.620^∗∗^	−0.181^∗∗^	0.121^∗∗^	1					
Job resources	4.480	0.810	−0.060^∗^	−0.034	0.096^∗∗^	−0.077^∗∗^	0.106^∗∗^	−0.052^∗^	−0.110^∗∗^	−0.089^∗∗^	1				
Job demands	3.243	0.841	0.012	0.039	−0.063^∗^	0.053^∗^	−0.058^∗^	0.118^∗∗^	0.109^∗∗^	−0.013	−0.434^∗∗^	1			
Confucian coping	4.572	0.855	−0.073^∗∗^	−0.061^∗^	0.070^∗∗^	−0.048	0.073^∗∗^	−0.074^∗∗^	−0.050^∗^	−0.032	0.495^∗∗^	−0.417^∗∗^	1		
Job engagement	4.154	1.056	−0.108^∗∗^	−0.045	0.135^∗∗^	−0.079^∗∗^	0.141^∗∗^	−0.055^∗^	−0.121^∗∗^	−0.073^∗∗^	0.543^∗∗^	−0.553^∗∗^	0.457^∗∗^	1	
Job burnout	3.128	1.174	0.059^∗^	0.024	−0.051^∗^	0.107^∗∗^	−0.092^∗∗^	0.095^∗∗^	0.085^∗∗^	0.068^∗∗^	−0.603^∗∗^	0.596^∗∗^	−0.473^∗∗^	−0.573^∗∗^	1

*Note:* M = mean value; gender (1 = male, 2 = female); education level (1 = high school degree, 2 = bachelor’s degree or tertiary college, 3 = master’s degree, 4 = doctoral degree); years of work experience (1 = less than 5 years, 2 = 5–9 years, 3 = 10–15 years, 4 = 16–20 years, 5 = more than 20 years); position (1 = non‐nurses, 2 = nurses); professional title (1 = no title, 2 = primary, 3 = intermediate, 4 = subsenior, 5 = senior); weekly working hours (1 = less than 40 h, 2 = 40–49 h, 3 = 50–59 h, 4 = 60–69 h, 5 = 70–79 h, 6 = more than 80 h); night‐shift frequency per week (1 = none, 2 = 1–2 times, 3 = 3–4 times, 4 = more than 5 times); employment method (1 = regular, 2 = contract).

Abbreviation: SD, standard deviation.

^∗^ *p* < 0.05.

^∗∗^ *p* < 0.01.

### 4.3. Validation of the Hypothesised Model

**FIGURE 3:**
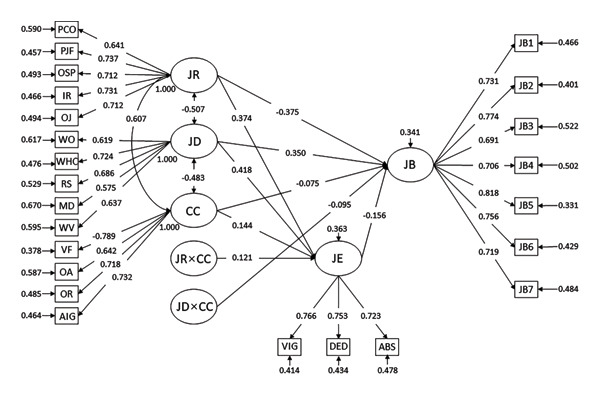
Standard error of mean analysis results of the moderating effect of Confucian coping on the relationship between job resources and job engagement, and between job demands and job burnout. Abbreviations: JB, job burnout; JD, job demands; JE, job engagement; JR, job resources; PCO, perceived career opportunity; PJF, person–job fit; OSP, organisational status perception; IR, interpersonal relationships; OJ, organisational justice; WO, work overload; WHC, work–home conflict; RS, research stress; MD, mental demand; WV, workplace violence; CC, Confucian coping; OA, optimism in the adversity; VF, viewpoints on ‘fate’; RH, responsibility as human beings; AIG, the role of adversity in individual growth.

We defined a latent variable moderation model with job demands and resources as independent variables, Confucian coping as the moderator, and job engagement/burnout as the outcomes (Table 3; Figure 3). The results of confirmatory factor analysis were as follows: *χ* ^2^ = 1106.017, df = 142, CFI = 0.955, TLI = 0.946, RMSEA = 0.064, SRMR = 0.046. Although the normalised chi‐square (*χ* ^2^/df) value of 7.789 did not satisfy the fit criterion, the other indicators met the requirements. Therefore, the model was considered to fit well. The larger value of *χ* ^2^/df was due to the larger sample size [64].

**TABLE 3:** The moderating effect of Confucian coping on the relationship between job resources and job engagement, and between job demands and job burnout.

Path	Total (*n* = 1653)		Nurses (*n* = 960)		Non‐nursing (*n* = 693)	
	*β*	95% CI	*β*	95% CI	*β*	95% CI
JD ⟶ JE	−0.418^∗∗∗^	[−0.474, −0.362]	−0.477^∗∗∗^	[−0.382, −0.180]	−0.281^∗∗∗^	[−0.545, −0.410]
JR ⟶ JE	0.374^∗∗∗^	[0.311, 0.437]	0.399^∗∗∗^	[0.205, 0.407]	0.306^∗∗∗^	[0.320, 0.478]
CC ⟶ JE	0.144^∗∗∗^	[0.079, 0.209]	0.085^∗^	[0.181, 0.420]	0.301^∗∗∗^	[0.010, 0.160]
JR × CC ⟶ JE	0.121^∗∗∗^	[0.077, 0.164]	0.109^∗∗∗^	[0.075, 0.214]	0.144^∗∗∗^	[0.056, 0.163]
JD ⟶ JB	0.35^∗∗∗^	[0.289, 0.411]	0.361^∗∗∗^	[0.203, 0.402]	0.302^∗∗∗^	[0.281, 0.442]
JR ⟶ JB	−0.375^∗∗∗^	[−0.438, −0.312]	−0.367^∗∗∗^	[−0.468, −0.268]	−0.368^∗∗∗^	[−0.448, −0.287]
CC ⟶ JB	−0.075^∗^	[−0.133, −0.017]	−0.053	[−0.266, −0.031]	−0.149^∗^	[−0.118, 0.011]
JD × CC ⟶ JB	−0.095^∗∗∗^	[−0.132, −0.058]	−0.097^∗∗∗^	[−0.143, −0.011]	−0.077^∗^	[−0.140, −0.053]
JE ⟶ JB	−0.156^∗∗∗^	[−0.234, −0.078]	−0.202^∗∗∗^	[−0.200, 0.038]	−0.081	[−0.309, −0.096]

*Note: β = *standardised estimate; non‐nursing (doctors and pharmacists/technicians).

Abbreviations: CC, Confucian coping; JB, job burnout; JD, job demands; JE, job engagement; JR, job resources.

^∗^ *p* < 0.05.

^∗∗^ *p* < 0.01.

^∗∗∗^ *p* < 0.001.

**FIGURE 4:**
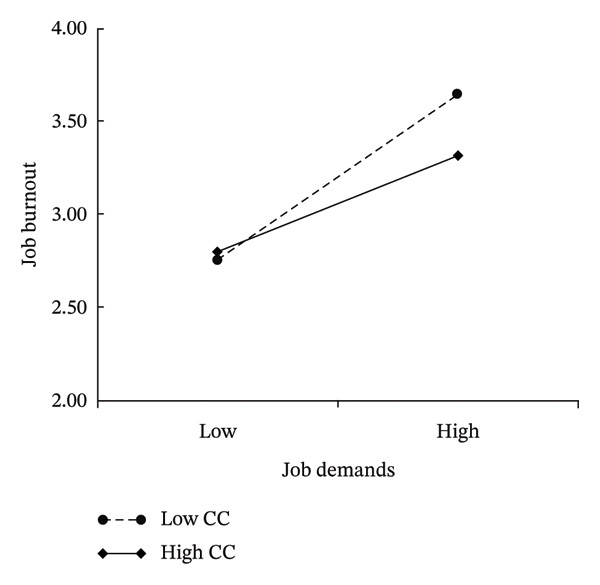
Diagram of the moderating effect of Confucian coping on the relationship between job demands and job burnout.

The analysis revealed that job demands had a significant positive effect on job burnout (*β* = 0.350, 95% CI [0.289, 0.411], *p* < 0.001) and a negative effect on job engagement (*β* = −0.418, 95% CI [−0.474, −0.362], *p* < 0.001), supporting H1 and H2. Notably, Confucian coping demonstrated both a negative association with job burnout (*β* = −0.075, 95% CI [−0.133, −0.017], *p* < 0.001) and a significant negative moderating effect on the job demands–job burnout pathway (*β* = −0.095, 95% CI [−0.132, −0.058], *p* < 0.001), confirming H5. This buffering role is visualised in Figure 4, where the slope of the job demands–job burnout relationship was steeper in the low Confucian coping group than in the high Confucion coping groups. This indicates that heightened Confucian coping attenuated burnout risk under escalating job demands.

**FIGURE 5:**
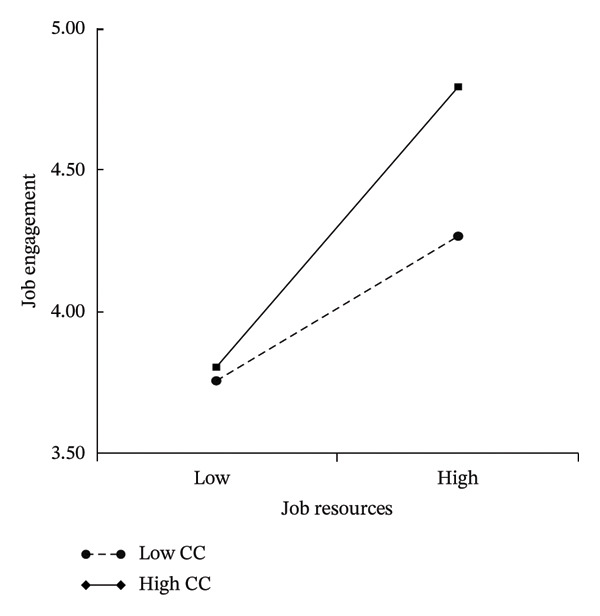
Diagram of the moderating effect of Confucian coping on the relationship between job resources and job engagement.

Conversely, job resources positively predicted job engagement (*β* = 0.374, 95% CI [0.311, 0.437], *p* < 0.001) and negatively predicted job burnout (*β* = −0.375, 95% CI [−0.438, −0.312], *p* < 0.001), validating H3 and H4. Confucian coping not only enhanced job engagement (*β* = 0.144, 95% CI [0.079, 0.209], *p* < 0.001) but also positively moderated the relationship between job resources and engagement (*β* = 0.121, 95% CI [0.077, 0.164], *p* < 0.001), supporting H6. As depicted in Figure 5, the resources–engagement slope was significantly steeper for the high Confucian coping groups than for the low Confucian coping groups. This demonstrates that Confucian coping amplified the motivational potential of job resources.

To further discover whether Confucian culture has different influences on nurses and other medical staff, we tested the model using data from nurses (*n* = 960) and non‐nurses (doctors and pharmacists/technicians combined, *n* = 693) (Table 3). The model demonstrated acceptable fit in both groups (e.g., CFI/TLI > 0.90, RMSEA/SRMR < 0.08). Consistent with the overall results, the core moderating effects of Confucian coping were significant in both groups. It attenuated the positive relationship between job demands and burnout (nurses: *β* = −0.097, 95% CI [−0.143, −0.011], *p* < 0.001; non‐nursing: *β* = −0.077, 95% CI [−0.140, −0.053], *p* < 0.05) and strengthened the positive relationship between job resources and engagement (nurses: *β* = 0.109, 95% CI [0.075, 0.214], *p* < 0.001; non‐nursing: *β* = 0.144, 95% CI [0.056, 0.163], *p* < 0.001). However, the direct negative effect of Confucian coping on job burnout was significant only for non‐nurses (*β* = −0.149, 95% CI [−0.118, 0.011], *p* < 0.05), not for nurses (*β* = −0.053, 95% CI [−0.266, −0.031], *p* > 0.05). While job engagement served as a significant negative predictor of burnout for nurses (*β* = −0.202, 95% CI [−0.200, 0.038], *p* < 0.001), this direct protective pathway was not statistically significant for non‐nurses (*β* = −0.081, 95% CI [−0.309, −0.096], *p* > 0.05).

### 4.4. Sensitivity Analysis

To assess the robustness of the primary findings and control for the influence of potential confounding factors, a sensitivity analysis was conducted by re‐estimating the model with additional covariates. This approach allowed us to examine whether the core associations remained stable after adjusting for key demographic and work‐related variables. The adjusted model included gender, education level, years of work experience, job position, professional title, weekly working hours, night‐shift frequency per week, and employment method as control variables.

As shown in Table 4, the significance and direction of the core structural paths remained largely unchanged compared with those of the unadjusted model (Table 3). All key paths retained statistical significance and no sign reversals were observed after including the control variables. These results suggest that the main associations identified in the primary analysis were robust and not substantially influenced by the sociodemographic factors considered, supporting the model’s stability and reliability.

**TABLE 4:** Results of sensitivity analysis.

Path	Total (*n = *1653)		Nurses (*n = *960)		Non‐nursing (*n = *693)	
	*β*	95% CI	*β*	95% CI	*β*	95% CI
JD ⟶ JE	−0.424^∗∗∗^	[−0.481, −0.368]	−0.289^∗∗∗^	[−0.392, −0.186]	−0.479^∗∗∗^	[−0.547, −0.411]
JR ⟶ JE	0.365^∗∗∗^	[0.301, 0.429]	0.296^∗∗∗^	[0.194, 0.399]	0.396^∗∗∗^	[0.316, 0.476]
CC ⟶ JE	0.136^∗∗∗^	[0.071, 0.201]	0.288^∗∗∗^	[0.167, 0.410]	0.078^∗^	[0.002, 0.153]
JR × CC ⟶ JE	0.120^∗∗∗^	[0.076, 0.163]	0.144^∗∗∗^	[0.074, 0.214]	0.109^∗∗∗^	[0.054, 0.163]
JD ⟶ JB	0.343^∗∗∗^	[0.280, 0.405]	0.295^∗∗∗^	[0.193, 0.396]	0.351^∗∗∗^	[0.269, 0.432]
JR ⟶ JB	−0.371^∗∗∗^	[−0.434, −0.308]	−0.368^∗∗∗^	[−0.467, −0.268]	−0.359^∗∗∗^	[−0.441, −0.278]
CC ⟶ JB	−0.073^∗^	[−0.131, −0.015]	−0.141^∗^	[−0.259, −0.023]	−0.053	[−0.118, 0.011]
JD × CC ⟶ JB	−0.097^∗∗∗^	[−0.133, −0.060]	−0.082^∗^	[−0.148, −0.015]	−0.098^∗∗∗^	[−0.142, −0.055]
JE ⟶ JB	−0.166^∗∗∗^	[−0.245, −0.087]	−0.091^∗∗∗^	[−0.211, 0.029]	−0.221^∗∗∗^	[−0.329, −0.112]
Gender ⟶ JE	−0.090	[−0.138, −0.042]	−0.098^∗^	[−0.163, −0.034]	−0.039	[−0.093, 0.015]
Education level ⟶ JE	−0.048	[−0.097, 0.001]	−0.040	[−0.115, 0.035]	−0.040	[−0.093, 0.013]
Years of work experience ⟶ JE	0.042	[−0.017, 0.102]	0.077	[−0.019, 0.173]	0.019	[−0.056, 0.093]
Position ⟶ JE	0.016	[−0.040, 0.071]	—	—	—	—
Professional title ⟶ JE	0.060	[−0.007, 0.127]	0.041	[−0.059, 0.141]	0.078	[−0.001, 0.157]
Weekly working hours ⟶ JE	0.016	[−0.029, 0.062]	−0.043	[−0.113, 0.028]	0.061^∗^	[0.008, 0.114]
Night‐shift frequency per week ⟶ JE	−0.031	[−0.074, 0.012]	0.015	[−0.054, 0.084]	−0.042	[−0.098, 0.013]
Employment method ⟶ JE	0.012	[−0.044, 0.067]	0.032	[−0.045, 0.110]	0.001	[−0.064, 0.065]
Gender ⟶ JB	−0.020	[−0.062, 0.021]	−0.007	[−0.067, 0.052]	−0.035	[−0.080, 0.010]
Education level ⟶ JB	0.023	[−0.020, 0.065]	0.050	[−0.018, 0.118]	0.001	[−0.044, 0.045]
Years of work experience ⟶ JB	0.068^∗∗^	[0.018, 0.119]	0.106^∗^	[0.019, 0.193]	0.041	[−0.020, 0.103]
Position ⟶ JB	0.064^∗∗^	[0.017, 0.112]	—	—	—	—
Professional title ⟶ JB	−0.023	[−0.081, 0.034]	−0.036	[−0.127, 0.054]	−0.014	[−0.081, 0.052]
Weekly working hours ⟶ JB	0.052^∗^	[0.012, 0.091]	0.065^∗^	[0.001, 0.129]	0.062^∗∗^	[0.016, 0.108]
Night‐shift frequency per week ⟶ JB	−0.008	[−0.045, 0.029]	−0.036	[−0.098, 0.026]	0.004	[−0.042, 0.050]
Employment method ⟶ JB	0.034	[−0.013, 0.081]	0.045	[−0.026, 0.115]	0.008	[−0.045, 0.062]

*Note: β = *standardised estimate; non‐nursing (doctors and pharmacists/technicians); gender (1 = male, 2 = female); education level (1 = high school degree, 2 = bachelor’s degree or tertiary college, 3 = master’s degree, 4 = doctoral degree); years of work experience (1 = less than 5 years, 2 = 5–9 years, 3 = 10–15 years, 4 = 16–20 years, 5 = more than 20 years); position (1 = non‐nurses, 2 = nurses); professional title (1 = no title, 2 = primary, 3 = intermediate, 4 = subsenior, 5 = senior); weekly working hours (1 = less than 40 h, 2 = 40–49 h, 3 = 50–59 h, 4 = 60–69 h, 5 = 70–79 h, 6 = more than 80 h); night‐shift frequency per week (1 = none, 2 = 1–2 times, 3 = 3–4 times, 4 = more than 5 times); employment method (1 = regular, 2 = contract).

Abbreviations: CC, Confucian coping; JB, job burnout; JD, job demands; JE, job engagement; JR, job resources.

^∗^ *p* < 0.05.

^∗∗^ *p* < 0.01.

^∗∗∗^ *p* < 0.001.

## 5. Discussion

This study examined the role of Confucian coping as a moderator in the JD–R model among healthcare professionals in Chinese tertiary public hospitals. The results supported all hypotheses, confirming that job demands positively predict burnout and negatively predict engagement, whereas job resources have a protective function. More importantly, Confucian coping significantly moderated these relationships, buffering the impact of job demands on job burnout and enhancing the effect of job resources on job engagement. The moderating role of Confucian coping suggests that values such as optimism, responsibility and reframing adversity as opportunities provide healthcare professionals with cognitive and emotional strategies to mitigate work‐related strain. While the moderating role of Confucian coping was significant across all medical staff, subgroup analysis revealed nuanced differences between nurses and other professionals, offering deeper insights into targeted management.

### 5.1. Theoretical Contributions

Medical staff in our study showed higher scores in specific dimensions of Confucian coping, particularly ‘The Role of Adversity in Individual Growth’ and ‘Optimism in Adversity’. High agreement with items such as *‘When faced with setbacks, I always try to learn something from them’* and *‘When I am unlucky, I try to develop myself and prepare for the future’* reflects an internalisation of core Confucian principles that align with the spirit of Mencius’ famous saying, *‘Thus when Heaven is going to give a great mission to someone, it first makes his body and mind endure suffering.’* This pattern suggests that medical staff have naturally developed positive interpretive frameworks for professional challenges, potentially through both cultural socialisation and occupational experiences.

Another dimension with a relatively high score was ‘Responsibility as Human beings’. This dimension is operationalised through items that assess a fundamental belief in innate human goodness (e.g., rejection of statements such as ‘In modern society, moral character is unimportant’ and ‘It is hard to say that human nature is inherently good’) [43]. This establishes a specific philosophical foundation: the sense of responsibility is not merely an external role obligation but arises from the conviction that humans are born with a benevolent nature and thus naturally bear social responsibilities. Crucially, this innate tendency is realised by fulfilling role‐dependent obligations within ritual interactions, where individuals act as rites‐bearers rather than as rights‐bearers [65]. The medical staff in this study were from tertiary public hospitals with strong social welfare mandates and were required to have a strong sense of professional responsibility. Meanwhile, the Confucian ideal of ‘*Ren*’ (仁, benevolence) merges with the institutional mandate of tertiary public hospitals, creating the professional ethos of ‘*Ren Xin Ren Shu*’ (仁心仁术, benevolent heart, benevolent medical skill) [66]. Hence, the relatively high scores on this dimension may be attributed to the synergy between Confucian values and professional responsibility. This cultural–professional synergy may provide a more profound and resilient motivational structure than professionalism alone.

Conversely, the score on the dimension of ‘Viewpoints on Fate’ was significantly lower than the others. This indicates that the medical staff in this study tended to reject fatalistic explanations and emphasised their determination to make every effort to change the situation. Previous studies on Confucian coping among Chinese individuals have found that higher scores on this dimension correlate with increased rates of depression and anxiety, as well as with lower life satisfaction due to negative attitudes towards life and an aversion to exerting effort [43]. The understanding of ‘fate’ is complex and gradually changing in Confucian culture. Ancient Chinese people attributed ‘mysterious events’ that were beyond their understanding or control to fate. However, with the advancement of science and technology and the development of Chinese society, practical Confucian coping has increasingly stressed provoking one’s subjective initiative against fate [67]. Thus, based on the scores for this dimension, we argue that Confucian coping may become a driving force in the consciousness of medical staff routinely facing stressful and excessive workloads. However, the notably low score on ‘Viewpoints on Fate’ also presents a potential double‐edged sword. While it reflects a commendable emphasis on personal agency and effort, in extreme circumstances, it may predispose medical staff to excessive self‐blame or moral distress when faced with medically inevitable poor outcomes or systemic constraints beyond their control. Future research should explore this potential dark side by investigating how to foster the proactive aspects of Confucian coping while mitigating the risk of undue self‐reproach.

The subgroup analysis revealed that, while the core moderating role of Confucian coping was significant across both groups, its direct protective benefits diverged. Confucian coping directly reduced burnout only among non‐nurses, whereas job engagement emerged as a direct safeguard against burnout among nurses. This result suggests that, for nurses, who endure intense emotional and physical demands, the pathway to well‐being may depend less on individual resources acting alone and more on cultivating a state of profound work engagement. Therefore, for nurses, the key function of Confucian coping may lie in helping them reframe adversity and more fully utilise job resources, thereby fostering an engaged work state that directly protects against burnout.

These findings reinforce the importance of extending the JD–R model to the hospital sector, demonstrating its adequacy for understanding which resources and demands affect healthcare personnel the most. Introducing Confucianism into the model will enhance the importance of cultural variables in future studies related to the healthcare sector. Finally, to complement previous studies [23], this integration of cultural values (such as Confucianism) is a relevant theoretical contribution that expands the existing literature on the JD–R model and theoretical studies on nurse management.

### 5.2. Practical Contributions

Based on these findings, we propose a dual‐path intervention strategy that targets both individual psychological and organisational job resources.

Hospital administrators should proactively foster psychological resources by integrating Confucian wisdom into staff development programmes. For example, group reflection sessions, scenario‐based workshops and mindfulness training can help all staff, especially nurses who bear significant emotional labour, reframe challenges as opportunities for growth, thereby strengthening their capacity to cope with adversity.

Therefore, enhancing tangible job resources is imperative. Foundational to this is upholding organisational justice and creating clear career pathways for all professional groups. For nurses, whose engagement directly mitigates burnout, ensuring adequate job resources is essential to sustain this protective pathway. A critical intervention involves conducting systematic on‐site workload assessments to scientifically align medical staffing levels with patient care demands, directly addressing the primary drivers of work overload. Furthermore, optimising the working environment to dismantle career advancement bottlenecks is vital for sustaining the engagement of the nursing workforce.

Finally, the global relevance of our findings lies in the demonstrated synergy between cultural values and organisational support. For nursing management in multicultural settings, this study highlights the importance of recognising and validating the unique local cultural resources that staff may bring to their work, which may enhance their well‐being and professional integration. Moreover, the core principles of Confucian coping, such as finding growth in adversity and a profound sense of duty, resonate with universal medical values. Thus, interventions inspired by these principles could be adapted in diverse cultural settings to strengthen resilience and engagement among medical staff.

### 5.3. Limitations and Future Research

As this study used a cross‐sectional design, causal inferences could not be made, and the statistical significance of the moderating function of Confucian coping requires practical examination. Considering the universality of Confucian culture in China, this study was primarily designed to cover all frontline clinical medical staff professions. Although the consistency of Confucian coping’s moderating role in overall results and subgroups reflected that universality, we still believe that Confucian coping’s dimension‐ and profession‐specific impacts should be further explored; for instance, longitudinal studies could be conducted in the future to gain deeper insight into the effect of Confucian coping’s certain dimension (such as ‘View of fate’) on the working status of nurses. Additionally, all data were collected from Guizhou Province, China, which has a relatively lower economic development level and limited healthcare resources compared with the more developed eastern provinces. Furthermore, the use of self‐reported measures may introduce common method and social desirability biases. These factors may influence the manifestation of Confucian coping and its interaction with job demands and resources, potentially limiting the direct applicability of these findings to more affluent regions in China. Therefore, future studies should adopt multisource and multiprovincial sampling strategies to enhance external validity. Comparative studies across diverse regions are recommended to determine the moderating roles of regional, cultural and economic factors.

## 6. Conclusion

This study provides novel insights into the influence of Confucian coping strategies on medical staff in Chinese tertiary public hospitals. Confucian coping appeared to attenuate the negative impact of job demands on burnout and strengthen the positive influence of job resources on engagement, which may serve as a meaningful personal resource in high‐demand healthcare environments.

## Funding

This study was supported by the Science and Technology Fund of the Guizhou Provincial Health Commission gzwkj2025‐174, Guizhou Provincial People’s Hospital Talent Fund Project [2023]‐26, and the Fundação para a Ciência e a Tecnologia (FCT) Strategic Project UIDB/00315/2020.

## Conflicts of Interest

The authors declare no conflicts of interest.

## Supporting Information

This study includes the following Supporting Information. Supporting File 1 contains the STROBE checklist for the reporting of observational studies.

## Supporting information


**Supporting Information** Additional supporting information can be found online in the Supporting Information section.

## Data Availability

The data supporting this study’s findings are available upon reasonable request from the corresponding author. The data are not publicly available due to privacy and ethical restrictions.
